# Translation from english into Urdu of a clinical decision tool to screen older women with back pain for osteoporotic-related vertebral fragility fractures

**DOI:** 10.1186/s12891-025-08837-z

**Published:** 2025-07-18

**Authors:** Tanzeela Y. Khalid, Huzaifa Adamali, Najma Zahoor, Sarah Drew, Emma M. Clark

**Affiliations:** 1 Bristol Medical School, University of Bristol, Bristol, UK; 2 North Bristol NHS Trust, Bristol, UK; 3 Musculoskeletal Research Unit, Learning and Research Building, Southmead Hospital, Bristol, BS10 5NB UK; 4 Lay member of the public, Bristol, UK

**Keywords:** Osteoporosis, Screening, Vertebral fracture, Urdu, Translation

## Abstract

**Background:**

Vertebral fractures associated with osteoporosis are common in individuals over 65, yet most cases go undiagnosed. This is concerning as those with vertebral fractures are at an increased risk of future fractures. To address this gap in healthcare, and facilitate diagnosis, so that bone-protective medications can be initiated, we developed and validated a clinical decision tool called ‘Vfrac’. Vfrac assists healthcare professionals in determining whether an older person with back pain requires a spinal radiograph to diagnose vertebral fractures. However, because Vfrac was developed in English, it remains inaccessible to non-English speakers. Given the high prevalence of vertebral fractures in South Asians, our study aimed to translate Vfrac from English to Urdu using a cross-cultural adaptation approach based on a standardised framework.

**Methods:**

Vfrac was translated into Urdu by five independent translators who were provided with information about the tool’s purpose and its components. Five different translators then back-translated the Urdu versions into English. A group of bilingual healthcare professionals and members of the public who spoke both English and Urdu reviewed and discussed the translations in reference to the original English questionnaire. The group selected the translations that best conveyed the intended meaning of each question, producing a final Urdu version of Vfrac. A bilingual healthcare professional conducted a final review and audio-recorded the Urdu Vfrac for accessibility.

**Results:**

The group of bilingual adults consisted of two healthcare professionals and three lay members, of whom four were female, and all aged 25 to 69 years. The final translated version of Vfrac written in Urdu was produced, ensuring it accurately reflected the original English version’s meaning. To improve accessibility, an audio-recorded version was also created.

**Conclusions:**

This study successfully adapted an English language clinical checklist, Vfrac, into Urdu through a structured cross-cultural adaptation process. To enhance accessibility and address healthcare disparities we strongly recommend incorporating audio-recordings alongside written translations.

**Study registration:**

This study received ethical approval by the Faculty of Health Sciences Research Ethics Committee (FREC Ethics Ref: 13801) on the 27th of April 2023.

**Supplementary Information:**

The online version contains supplementary material available at 10.1186/s12891-025-08837-z.

## Abstract (plain language summary)

Breaking a bone in the back (vertebral fracture) is common in people over 65. It’s often caused by osteoporosis, a condition that causes bones to weaken and break more easily. Identifying broken bones in the back enables healthcare professionals to prescribe bone strengthening medicines to reduce the risk of future breaks. However, many individuals with vertebral fractures remain undiagnosed.

We have previously worked with individuals who live with osteoporosis and back pain to develop a checklist named ‘Vfrac’. This checklist helps assess whether an older person with back pain is likely to have a vertebral fracture and whether they require a spinal x-ray for confirmation. Vfrac consists of 12 questions and three physical measurements, and is written in English. However, approximately 270,000 older adults in England and Wales speak Urdu as their primary language. This study followed a standardised method to translate Vfrac into Urdu while ensuring that the meaning remained intact rather than applying a direct word-for-word translation. To achieve this, five qualified translators first translated Vfrac to Urdu. Next, five different translators back-translated each Urdu version into English. A group of individuals from the target population then reviewed and compared the original Vfrac with the Urdu translations to develop a final, agreed-upon Urdu version. A bilingual co-author then checked the final version to ensure it made sense before audio-recording Urdu-Vfrac.

**Supplementary Information:**

The online version contains supplementary material available at 10.1186/s12891-025-08837-z.

## Introduction

Osteoporosis and related vertebral fragility fractures (VFFs) are common musculoskeletal conditions among older people affecting more than half of women and one fifth of men aged 50 years and above. However less than one third of cases receive clinical attention. VFFs often go undiagnosed due to multiple factors, including the high prevalence of general back pain in older people [1, 2], insufficient clinical knowledge regarding appropriate diagnostic triggers for spinal radiographs [3], unclear radiologist reporting of spinal X-rays, and inconsistent coding of spinal radiograph results in primary care [4]. Identifying VFFs is crucial for initiating appropriate osteoporosis treatment, based on local/national guidelines, ultimately reducing future fracture risks, which can lead to significant morbidity and disability.

To address this issue, Vfrac was developed as a clinical decision tool to help healthcare professionals determine whether an older person (aged 65 and over) with back pain requires a spinal radiograph to diagnose a VFF. Vfrac was developed using the Medical Research Council (MRC) framework for development and evaluation of complex interventions. Previous validation studies have demonstrated that Vfrac identifies 93% of adults with multiple vertebral fracture and two-thirds with a single vertebral fracture based on an algorithm-based qualitative approach to identification of VFFs [5]. The tool comprises 12 questions and three physical measurements [5] and can be administered in-person by healthcare professionals or through a hybrid approach where patients complete the questionnaire at home before undergoing physical measurements at a clinic visit [manuscript submitted].

Since the original Vfrac was developed in English, its accessibility is limited for non-English speakers. In England and Wales, English is the primary language for 91.1% of the population [6]. However, a significant proportion of the population speak other languages, with Urdu being one of the most spoken South Asian languages. Ensuring that Vfrac is available in Urdu is essential to improving accessibility for older adults who primarily communicate in their native language.

Although translation services are available in primary care, they are not always easily accessible and may extend consultation times. While artificial intelligence tools offer literal translations, they often fail to account for cultural differences, which can significantly affect how individuals express and experience pain [7–9]. Additionally, some patients may struggle with reading or writing in their preferred language, making oral translations essential for healthcare communication.

A standardized guideline for cross-cultural adaptation of clinical measures has been developed, incorporating methodological frameworks and socio-linguistic research principles [10]. This study follows this guideline to translate the Vfrac clinical decision tool from English to Urdu, ensuring linguistic and cultural relevance.

## Methods

### Study design

A cross-cultural adaptation study of a clinical decision tool.

The aims of this study were to use the cross-cultural adaptation approach to translate the Vfrac questionnaire plus its two possible outcomes (the need, or not, for a spinal radiograph/X-ray), into Urdu and to assess the quality and ease of use of the translation. The cross-cultural adaptation approach to translating health-related clinical questionnaires was followed [10] and has two components: a literal translation of individual words and sentences from one language to another; followed by an adaptation of the sentences with regard to idiom, cultural context and lifestyle. The quality and sensibility of the translation were then sense checked.

### Ethics approval

This study received approval from the University of Bristol’s Faculty of Health Sciences Research Ethics Committee (FREC Ethics Ref: 13801).

### Translation of Vfrac from english to Urdu

We collaborated with Universal Language solutions Ltd (London, United Kingdom) for translations. The original Vfrac was translated from English into Urdu by five separate linguists to produce five versions of the Vfrac questionnaire in Urdu. All five linguists were provided with a lay summary of the overall intent of Vfrac and the meaning of each component so that they are aware of the tool’s objectives, thus ensuring a more reliable restitution of the intended measure [11, 12]. Five additional linguists then back-translated these versions into English; they were not told of the underlying intent so were free of biases and expectations [10]. This process helped identify translation errors, ambiguities, and cultural inconsistencies.

### Recruitment of bilingual healthcare professionals and members of the public

A group of individuals representing the target population undertook a collaborative review of the original tool, and the forward and backward translations in order to produce a final modified version. The group included bilingual healthcare professionals and members of the public who spoke both English and Urdu fluently, with some of these individuals also knowledgeable in the disease area to ensure that idiomatic expressions and cultural nuances were appropriately reflected in the final version [13].

Healthcare professionals were identified via the North Bristol NHS Trust BAME (Black, Asian, and Minority Ethnic) Network, leveraging established community networks and connections. In addition, snowball sampling and outreach through community organisations like the Shahporan Islamic Centre and Mosque, and the Pakistan Women’s Association, were used to identify and recruit bilingual members of the public [14]. Clear, accessible study materials (including an invitation letter, information leaflet, and consent forms) were distributed, and reminder packs were sent to those who expressed interest but hadn’t followed through with consent forms. All participants were over 18 years of age and participants self-assessed their fluency in English and Urdu, expressing confidence that they had a level of competence required for the study. Informed written consent was obtained from 8 bilingual English and Urdu speakers prior to their participation in the group work.

This included four medical practitioners (doctors), an NHS research compliance manager, a retired midwife, and two lay members of the public. However, due to limited time availability, three of these bilingual speakers were unable to attend the consensus group work.

### Consensus group work to produce final translation of Vfrac

Two bilingual medical practitioners commented on the translated versions of Vfrac beforehand to reduce the list of suitable translations down to two or three, instead of five. However, all five translations were still made available as paper copies at the group meeting for discussion.

Two bilingual healthcare professionals and three members of the public (4 female; 1 male; within age group categories from 25 to 69 years) attended a group meeting with authors (TK and EC) to review and discuss the five Urdu translations in reference to the original questionnaire in English. The meeting was conducted face-to-face over a 2-hour period.

An introduction on why Vfrac was developed was given by the Vfrac developer (EC) and thereafter an explanation of the meaning behind each Vfrac question in English. Participants were given time to read each Vfrac component, the five translations, to discuss amongst themselves in English or Urdu, and ask any questions. Thereafter the group reviewed the five translations to identify which most closely represented the intended meaning of each question. To check for experiential and conceptual equivalence between the source Vfrac and the final translation, group members were probed to explain their understanding of each question in an open-ended manner and to detect discrepancies [15].

Where there was no single best translation for a question, one or more of the current translations were revised with the group to formulate an accurate translation of the original question in English. Answer options to each question were also translated into Urdu. To produce a translated version comprehensible to most people, simple language was used to meet a target reading age of 11. For sense-checking, the final translation of each component was read out loud to ensure the translation adequately reflected the original English-language material. Group members commented if they felt it gave rise to ambiguity or misinterpretation. Full consensus was achieved on each question translation before moving onto the next. Finally, the two possible result outcomes of Vfrac were also translated. Each participant was given a thank you card containing a £25 shopping voucher to thank them for their time.

### Final sense checking and audio-recording of Urdu-Vfrac

The recording of the Urdu-Vfrac was conducted following consensus group work. A bilingual retired healthcare professional (NZ) who attended the group work facilitated the audio recording. Audacity software (version 3.4.2, Boston, MA, USA) was used for all audio recordings. Before recording, the Urdu-Vfrac was read aloud to check for grammatical errors and to ensure clarity in spoken content. There was also agreement on the spoken content for each audio file, ensuring adequate pauses between each question-and-answer option for ease of use and future integration within the web-based tool. Minor grammatical changes were made to the text if necessary and a 3-second pause was added between each question-and-answer option. Each component of the Vfrac tool was recorded as a separate audio file and was saved in the lossless waveform audio (WAV) file format to maintain good preservation of audio quality. The recordings took place in a quiet room at the University of Bristol, where NZ wore a Blackwire 3320 headset (Plantronics, Mexico) to prevent sound bleed and ensure clear audio-recordings. Each audio recording was played back to check clarity and sound quality. Finally, the recordings were saved on a restricted-access university drive, available only to the research team.

## Results

The focus of the group work was to ensure that translations were clear and culturally appropriate. Some key observations following the group work have been summarised below.

### Descriptive versus simple translations

Simplification of language was a key consideration. For example, instead of using a more complex phrase like “What is the measurement of your height today?“, a simpler version “How tall are you today?” was selected. This approach helped ensure the language was straightforward and easily understood by most people.

**Challenges with specific medical terminology**: Translating technical terms, such as “tragus,” posed challenges as there were concerns that overly detailed descriptions might confuse or worry the patient. To address this, a more indirect phrasing was chosen: “We want to measure the distance between your ear and the wall.” This kept the translation simple and clear while still conveying the necessary information. This particular measurement was to be taken by a healthcare professional during a face-to-face appointment so it was not deemed necessary to go into detail explaining the tragus.

### Cultural sensitivity in question phrasing

The translation process also took into account cultural norms. For example, when asking someone’s age, the literal translation of “How old are you?” was not preferred because it did not match how the question would be naturally asked in Urdu. Instead, the culturally appropriate phrasing “What is your age?” was chosen.

### Flow and naturalness of Language

A key aspect of selecting the best translation was ensuring that the sentence not only conveyed the intended meaning but also sounded natural in the target language. For instance, the question about height at age 25 had multiple correct translations, but one was chosen because it had a smoother structure that fit the rhythm and norms of the Urdu language.

### Clarity over literal translations

Literal translations were sometimes avoided in favour of terms that would be clearer for patients. For example, using “used” instead of “taken” in reference to steroid tablets helped eliminate any ambiguity.

### Simplicity in medical terminology

The simpler term “broken” was selected over the more technical “fracture” because it would be more easily understood by the general public, avoiding the need for additional explanations.

### Preference for commonly spoken words

The use of simpler, more commonly spoken Urdu words over more formal or academic terms was preferred. This meant the language used would be accessible to most Urdu speakers, not just those with higher language proficiency.

### Nuanced descriptions for specific terms and questions

Certain terms, like “sharp,” “reclining,” or explaining “back pain when working in the kitchen,” required deeper discussions to find the most accurate and culturally appropriate translation. In contrast some other questions, such as the asking of age, required very little discussion to reach consensus on the best translation.

In summary, there was a need for balance between ensuring technical accuracy and ensuring cultural and linguistic relevance, with the aim of making the language as accessible and clear as possible for patients who speak Urdu as their main language.

**Fig. 1:**
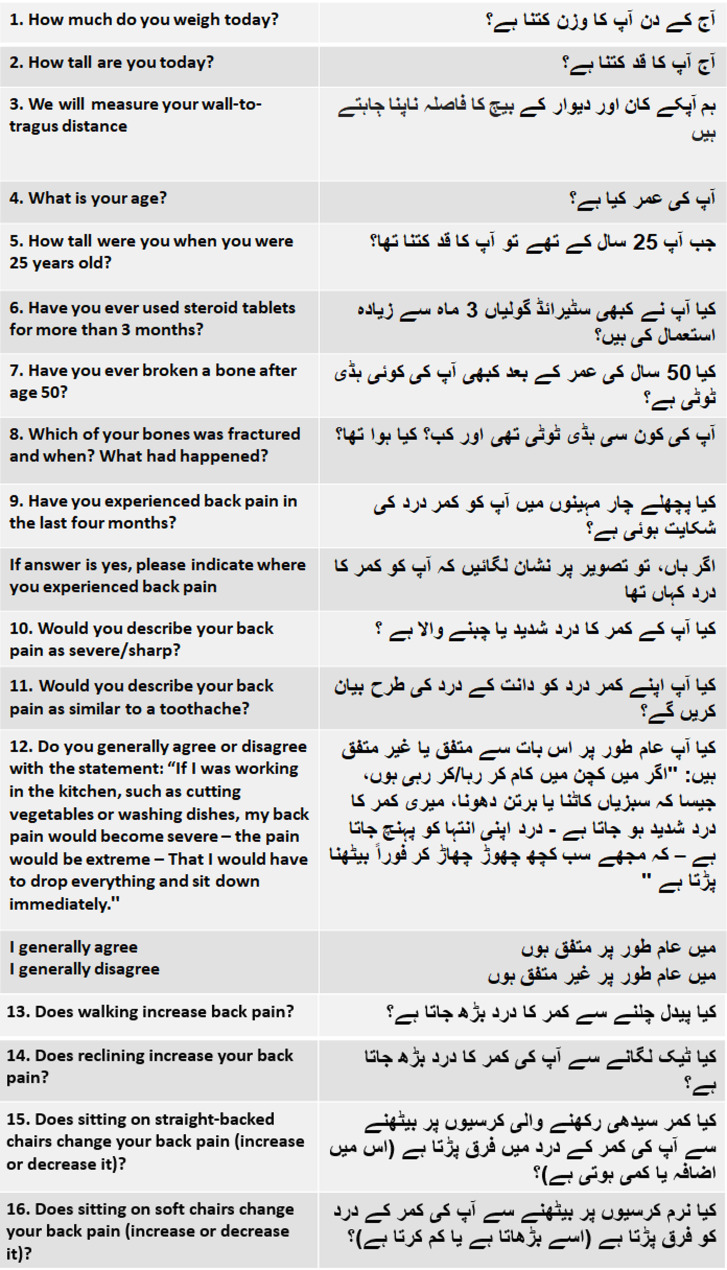
Urdu-Vfrac questions to help identify whether a spinal X-ray should be recommended for possible vertebral fragility fracture

**Fig. 2:**
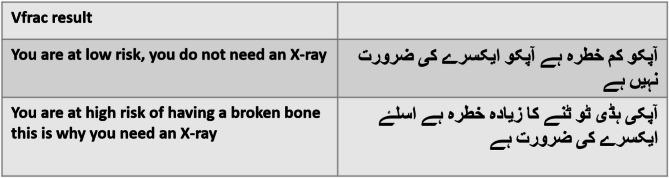
Urdu-Vfrac results outputs based on data entered into the Vfrac clinical decision tool

The final written Urdu-Vfrac is provided in Fig. 1 and a fuller version of Urdu-Vfrac including the answer options are provided in Supplementary file (1) The audio recording of Urdu-Vfrac is provided in Supplementary file (2) The two possible outputs of the Vfrac tool translated into Urdu (the need, or not, for a spinal radiograph/X-ray) are given in Fig. 2. Audio recordings of the low or high-risk result are provided in Supplementary file 3.

## Discussion

The translation of Vfrac into Urdu was a meticulous process that required attention to linguistic accuracy, cultural relevance and accessibility. Our approach ensured that the translated tool remained as effective as the original English version while being comprehensible to Urdu-speaking individuals. According to census data, there have been significant changes in the ethnic composition of England and Wales in recent years [16]. In 2001, 87.5% of the residents identified themselves as “White: English, Welsh, Scottish, Northern Irish or British”. In 2011, this figure was reduced to 80.5%, and in 2021, it further decreased to 74.4% [16]. The next most common ethnic group is “Asian, Asian British or Asian Welsh”, which accounts for 9.3% of the overall population. The largest increase in ethnic categories was observed in those who identified as Asian or “other ethnic group” [16]. Census 2021 data revealed that there were 270,000 residents living in England and Wales who spoke Urdu as their main language [6].

The translation of the Vfrac tool into Urdu and other widely spoken languages in the UK will help to minimise healthcare inequalities that may arise due to language barriers. This is particularly significant considering the UK’s 2010 Equality Act, which mandates the NHS to reduce any disparities in healthcare access. The guidelines for primary care, where the Vfrac tool will be utilized, state that “patients should be able to access primary care services without the language and communication barriers that may hinder them from receiving the same quality of healthcare as others” [17]. There must be a collaborative effort to make Vfrac and other clinical decision tools available in various languages, in order to keep up with the increasing pace of international migration. This will enable the tool’s application in other countries if it becomes available worldwide. It is crucial to ensure that ethnic minority patients who have limited English proficiency are not left behind and do not continue to receive substandard healthcare as this perpetuates healthcare inequalities.

Communities with language and cultural barriers are at an increased risk of limited health literacy which can widen health inequalities. Efforts to make the Vfrac tool ‘health-literacy friendly’ include the use of detailed instructions with labelled images to explain how the in-person physical measurements will be taken, and the provision of further information to explain what is meant by certain terms. For example, beneath the question: ‘Does reclining increase your back pain?’ There is the description: ‘Reclining is leaning back in a chair. Lying down does not count as reclining’. A paper version of the Vfrac questionnaire also exists for those who don’t have access to digital platforms. People from Black and South Asian communities often seek help with health information or surveys from credible and trustworthy members of their community (including local GPs, community groups), or young family members [18]. Therefore, it is important that healthcare professionals take a personalised approach when engaging with members from different cultural and language backgrounds to discuss and enable their individual preferences for the self-completion of questionnaires. In future, it is hoped that the online Vfrac tool (with translations) will be accessible for patients wishing to self-complete most questions at home prior to their face-to-face appointment where a healthcare professional will take their physical measurements.

Although we had a diverse group of participants, including healthcare professionals and lay individuals, there were some limitations to our study. The bilingual group only consisted of five people. However, 10 official translators had already been involved, and a group size of 5 meant that full discussion involving every member was possible. One of the main limitations was the small number of bilingual participants from an older age group. Only one participant was older than 65 years, which would have been more representative of the patient population that Vfrac is intended for. Additionally, although more men had expressed an interest in participating, they were unable to attend the group work. The original Vfrac tool (in English) was also tested in men with VFF, and qualitative interviews were undertaken to evaluate the wording of the Vfrac tool from the perspective of men [19]. This work found that the Vfrac tool had no gender-specific barriers, meaning it is applicable for use in men as well as women.

One of the most significant insights gained was the importance of accessibility beyond written translations. During the review process, it became evident that while many Urdu speakers could understand spoken Urdu, some could not read the script proficiently. To address this, we developed an audio-recorded version of Urdu-Vfrac, ensuring that individuals with low literacy levels could still benefit from the tool. The audio recordings were carefully reviewed to maintain accuracy in pronunciation and pacing, making them user-friendly for healthcare settings. The need for audio recordings highlights a broader issue in healthcare communication: many translated materials assume literacy proficiency in the target language. By incorporating both written and spoken Urdu versions of Vfrac, we took a step towards addressing these disparities and ensuring equitable access to healthcare tools for all Urdu-speaking individuals.

Vfrac has potential as a clinical tool in primary care, with previous research suggesting it is valid in older adults [5]. However, uncertainties remain around its real-world implementation and cost-effectiveness from the NHS perspective. We have recently completed feasibility work to conduct a cluster RCT for a definitive evaluation of the effectiveness of Vfrac in the real world. The future trial will assess Vfrac against standard care for older women with back pain from the NHS perspective. We will make the Urdu-Vfrac translation available in this future trial. Translations will be made available as both text and audio files through a separate webpage accessed via the online Vfrac tool.

## Electronic supplementary material

Below is the link to the electronic supplementary material.

Supplementary Material 1

Supplementary Material 2

Supplementary Material 3

## Data Availability

All data generated during this study is included in this published article and its supplementary information files.
