# Supplementary figures and images for: Translation from english into Urdu of a clinical decision tool to screen older women with back pain for osteoporotic-related vertebral fragility fractures
Source: BMC Musculoskelet Disord. 2025 Jul 18;26:691. doi: 10.1186/s12891-025-08837-z (PMC12273021; doi:10.1186/s12891-025-08837-z)

**Supplementary file 1: Urdu-Vfrac in full with answer options**


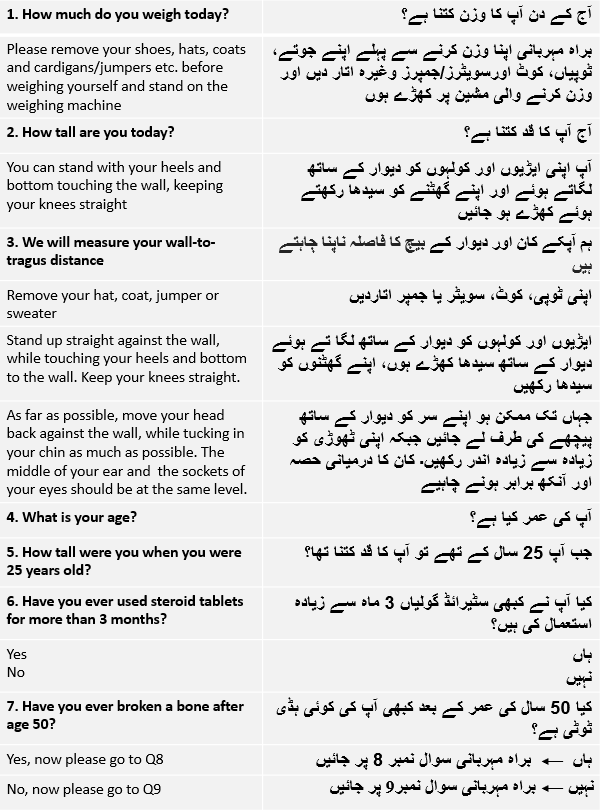


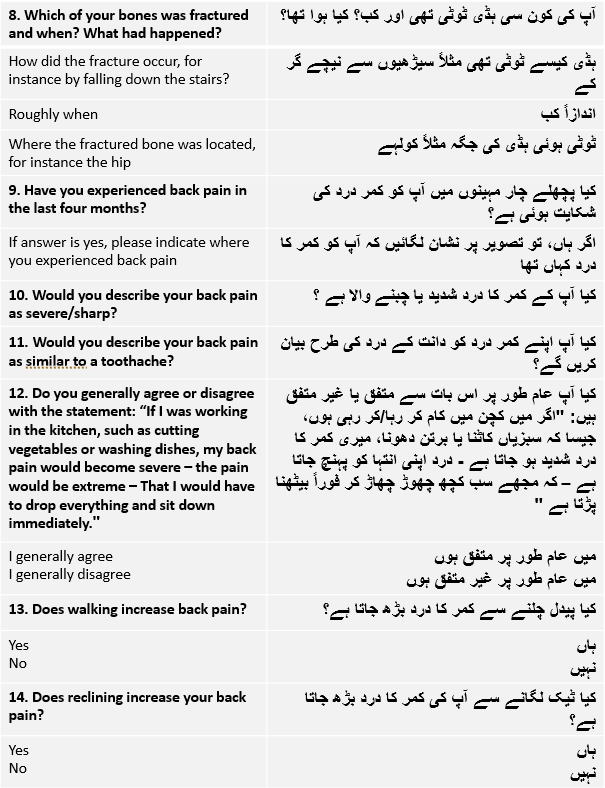


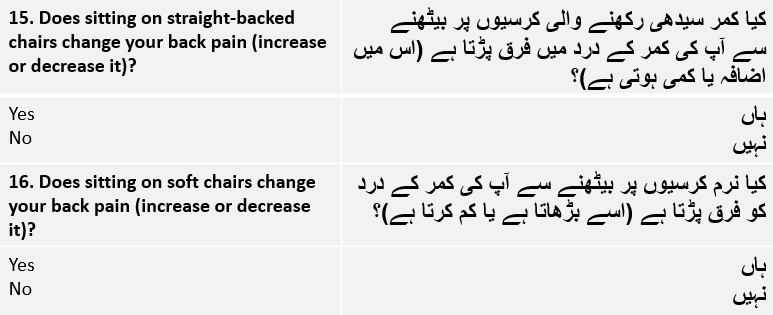

Supplement: Supplementary file 1 — Supplementary Material 1 [file 12891_2025_8837_MOESM1_ESM.docx]
